# The effect of regional disparities in access to rehabilitation services on mortality among stroke patients: A Korean nationwide population-based retrospective cohort study

**DOI:** 10.1097/MD.0000000000051192

**Published:** 2026-10-23

**Authors:** Dong-Yup Yoo, Chang-Yoon Baek, Jung-Kyu Choi

**Affiliations:** a Department of Rehabilitation Medicine, National Health Insurance Ilsan Hospital, Goyang, South Korea; b Health Insurance Research Institute, National Health Insurance Service, Wonju, South Korea.

**Keywords:** mortality, regional disparities, rehabilitation, stroke

## Abstract

This study aimed to analyze physical therapy (PT) and occupational therapy (OT) claims data to determine whether access barriers to rehabilitation services due to regional factors affect PT and OT accessibility. Furthermore, we investigated the mortality rate of stroke patients by region in South Korea using the National Health Insurance Service-National Sample Cohort database. This is a register-based, retrospective cohort study. Using data from the National Health Insurance Service database, we included patients who were newly diagnosed with stroke between 2005 and 2017. Of the 19,067 patients diagnosed with stroke, 2263 who died within 6 months were excluded. The final sample included 16,804 patients (51.3% men and 48.7% women). Residential regions were categorized into Capital, Capital area, Metropolitan, and Rural area. After adjusting for covariates, a logistic regression model was used to analyze the accessibility of rehabilitation services by residential region. The Kaplan–Meier method was employed to compare the survival rates of rehabilitation patients across different residential regions. A Cox proportional hazards model was used to estimate the independent contribution of each factor to mortality risk (hazard ratios [HRs] and 95% confidence interval [CIs]). Significant differences in mortality were observed according to sociodemographic factors and Charlson Comorbidity Index scores. Accessibility in Capital area and Metropolitan cities was comparable to the Capital. However, patients living in a rural area had significantly lower access to rehabilitation compared to those in the Capital (PT: OR = 0.889, 95% CIs: 0.809–0.987, *P* = .029; OT: OR = 0.802, 95% CIs: 0.726–0.886, *P* = .001). Notably, among those receiving inpatient rehabilitation, rural area residency was associated with a higher risk of death compared to the Capital (HRs = 1.288; 95% CIs, 1.015–1.635; *P* = .037). In contrast, no significant disparity was found in the outpatient group (HRs = 1.011; 95% CIs, 0.917–1.115; *P* = .820). Stroke patients living in rural area were less likely to receive rehabilitation services than those in the capital. This regional disparity in access, particularly in inpatient rehabilitation, is associated with an increased risk of long-term mortality after stroke. These findings suggest that improving inpatient rehabilitation infrastructure in rural area is critical to reducing survival gaps.

## 1. Introduction

Globally, stroke, a major chronic disease, was reported as the third most common disease and the fifth leading cause of death in 2019.^[1,2]^ Stroke survivors often require temporary or lifelong care, imposing a significant burden in terms of socioeconomic costs.^[3]^ Even when patients survive, many suffer from various functional impairments, such as physical disability, cognitive impairment, memory loss, and dysphagia.^[4,5]^ Furthermore, prolonged hospitalization in intensive care units following stroke onset is associated with complications, including pressure sores, physical and cognitive decline, depression due to sleep deprivation, poor communication and nutritional status, and restrictions on repositioning.^[6,7]^

Therefore, early rehabilitation, rather than traditional bed rest, is crucial for preventing complications and promoting the recovery of bodily functions, including speech and swallowing.^[4,8]^ Active rehabilitation services are essential for stroke patients to maximize physical recovery and eventually lead independent lives.^[9]^ Beyond physical recovery, rehabilitation serves as a vital process for regaining independence in daily activities and resuming social participation.^[10]^

Factors affecting the utilization of rehabilitation services among stroke patients include age, sex, race, disease severity, comorbidities, individual economic status, social security type, and regional accessibility.^[10,11]^ Among these, regional factors play a dominant role, as physical limitations and geographical barriers often restrict access to rehabilitation services.^[12]^ In many countries, certain regions – particularly rural area – lack professional medical institutions, making timely rehabilitation services unavailable.^[13]^ Due to disparities in accessibility between rural area and urban regions, individuals living in rural area are likely to experience difficulties in accessing medical services, which may increase the risk of delayed physical recovery, cognitive impairment, poor quality of life, complications, and depression.^[14,15]^

Because stroke units and convalescent rehabilitation hospitals are primarily concentrated in metropolitan areas,^[16]^ poststroke patients in rural area face significant barriers to rehabilitation services compared to those in urban areas.^[17]^ In the Republic of Korea, this regional imbalance is exceptionally pronounced, with more than half of the country’s specialized medical infrastructure and comprehensive rehabilitation centers clustered within the capital area.^[18]^ Previous research has indicated that patients in rural and suburban areas are significantly less likely to receive rehabilitation services due to these geographical barriers.^[19]^ However, empirical evidence regarding how such regional disparities in access to rehabilitation services directly affect mortality among stroke patients remains limited.

While prior studies have mostly focused on single-center observations or short-term in-hospital outcomes, evidence evaluating how the long-term survival benefits of rehabilitation are structurally compromised across geographic regions remains sparse. By leveraging a comprehensive nationwide cohort, this study addresses this key knowledge gap by analyzing the dual impact of regional disparities on both immediate service accessibility and long-term stroke survival, going beyond localized short-term observations.

The Republic of Korea provides ideal conditions for big data research, including stroke studies, as all citizens are enrolled in the National Health Insurance, and a unified insurance claims database is established. Furthermore, because rural-urban infrastructure disparities and centralized healthcare resources are common structural challenges, these national findings offer valuable insights for other healthcare systems worldwide operating under universal health coverage and facing geographic inequities. Therefore, this study aimed to analyze physical therapy (PT) and occupational therapy (OT) claims data to determine whether access barriers to rehabilitation services due to regional factors affect PT and OT accessibility. Furthermore, utilizing the National Health Insurance Service (NHIS)-National Sample Cohort (NSC) database, we elucidated how these geographical imbalances ultimately translate into disparities in mortality rates among both inpatient and outpatient stroke populations. Specifically, using nationwide long-term cohort data (2005–2017) of 16,804 stroke patients, we evaluated PT/OT accessibility and long-term mortality across 4 regional categories (Capital, Capital area, Metropolitan, and Rural area) with stratified analysis for inpatient and outpatient settings. Ultimately, our findings provide empirical evidence to guide public health policies, optimize regional healthcare resource allocation, and support future clinical research aimed at establishing geographic equity in poststroke rehabilitation.

## 2. Methods

### 2.1. Data extraction and study population

NHIS established a sample cohort of 1,000,000 patients who received health insurance coverage or medical benefits in 2006. This nationwide recording system, encompassing medical claims and evaluation data, provided a longitudinal cohort from 2002 to 2019.^[20,21]^ The medical treatment database comprises 57 variables containing comprehensive information on inpatient and outpatient care, procedures, and prescriptions. Diseases were classified according to the Korean Classification of Diseases and Causes of Death, which corresponds to the International Classification of Diseases, 10th Revision. For this analysis, we identified patients diagnosed with stroke (International Classification of Diseases, 10th revision codes I60–I64) between 2005 and 2017. To include only newly diagnosed cases, patients diagnosed between 2002 and 2004 were excluded (washout period). Additionally, patients from 2018 to 2019 were excluded to ensure a minimum follow-up period of at least 2 years. Of the 19,067 patients initially identified, 2263 who died within 6 months were excluded to focus on long-term survival and rehabilitation outcomes. The final sample included 16,804 patients (Fig. 1). The mean follow-up period was 7.3 ± 3.8 years for survivors and 5.2 ± 3.6 years for non-survivors. All study components and procedures were approved by the Institutional Review Board of the National Health Insurance Service Ilsan Medical Center (Approval No. NHIMC-2021-05-010). We utilized the NSC data (Data No. NHIS-2022-2-116) provided by the NHIS. As this study used de-identified secondary data, the requirement for informed consent was exempted.

**Figure 1.**
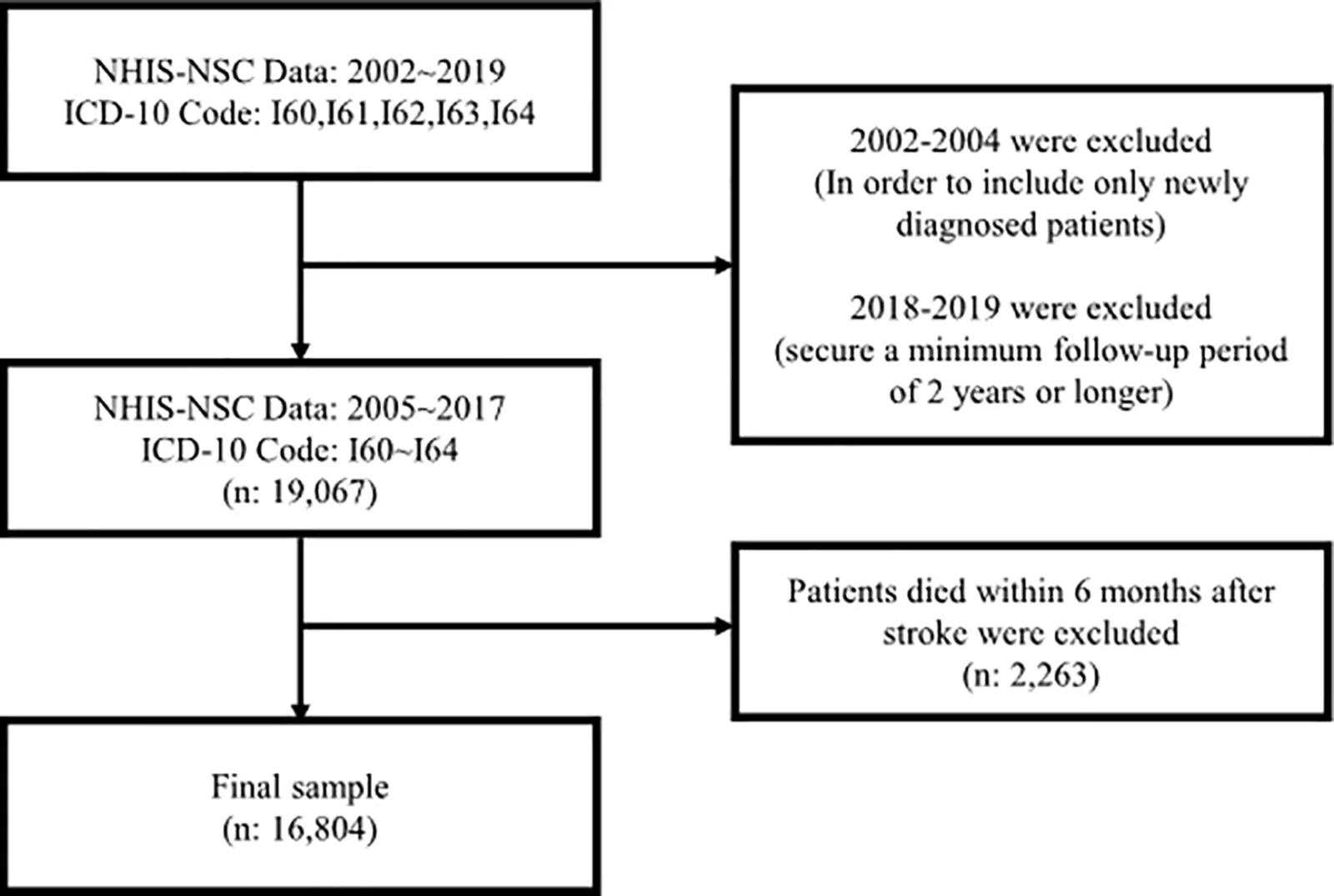
Flowchart of study population selection process. Out of 19,067 patients diagnosed with stroke between 2005 and 2017 in the NHIS-NSC database (2002–2019), patients diagnosed between 2002 and 2004 were excluded in order to include only newly diagnosed patients. Patients diagnosed between 2018 and 2019 were excluded to secure a minimum follow-up period of 2 years or longer. Additionally, patients who died within 6 months after stroke onset were excluded (n = 2263), resulting in a final sample of 16,804 patients. ICD-10 = International Classification of Diseases, 10th revision, NHIS = National Health Insurance Service, NSC = National Sample Cohort.

### 2.2. Study variables

In Korea, medical services prescribed to patients are billed to the Health Insurance Review and Assessment Service and recorded in the NHIS database. PT and OT were classified according to the codes claimed during rehabilitation. PT included specialized therapy for central nervous system disorders (MM105), mattress or mobilization training (MM301), and gait training (MM302). OT included complex OT (MM112), special OT (MM113), activities of daily living training (MM114), and rehabilitative dysphagia therapy (MX141). Specifically, MM105 was billed when a rehabilitation specialist or a physical therapist with > 120 hours of specialized training (e.g., Vojta or Bobath therapy) conducted a one-on-one session for > 30 minutes to treat muscle paralysis or stiffness due to central nervous system disorders. MM301 was used for patients with limited mobility (e.g., amputation, hemiplegia, or cerebral palsy) who received mattress, mobility, or chair training for > 30 minutes. MM302 was applied when patients with gait impairments due to central nervous system diseases received gait training for > 30 minutes. Regarding OT, MM112 (10 minutes) focuses on targeted upper extremity function and basic task performance, whereas MM113 (> 30 minutes) involves intensive rehabilitation for severe cognitive, perceptual, or neurodevelopmental deficits. MM114 was billed for one-on-one treatments lasting > 20 minutes. MX141 was used for one-on-one dysphagia training (> 30 minutes) for patients with disorders of the central nervous system, esophagus, or airways.^[22]^ While other specialized rehabilitation modalities (e.g., speech-therapy, cognitive rehabilitation, and mental health counseling) are also recorded in the database, the present study specifically focused on PT and OT. These modalities represent the primary standardized interventions directly targeted at restoring physical mobility, activities of daily living, and swallowing function, which are pivotal physical determinants of poststroke functional recovery and long-term survival.

### 2.3. Regional classification

The regional classification criteria were defined based on the Korean Local Autonomy Act, population size, and the administrative districts where specialized stroke units and convalescent rehabilitation medical institutions are concentrated.

Seoul, the capital of the Republic of Korea, was designated as the Capital (reference region) due to the high concentration of both stroke treatment rooms (Fig. 2A) and convalescent rehabilitation medical institutions (Fig. 2B). Incheon Metropolitan City and Gyeonggi Province were defined as the Capital area. Other major cities (Busan, Daegu, Daejeon, Gwangju, Ulsan, and Changwon) with a population of 1 million or more outside the Capital area were defined as Metropolitan, and all other regions were categorized as rural area (Fig. 3).

**Figure 2.**
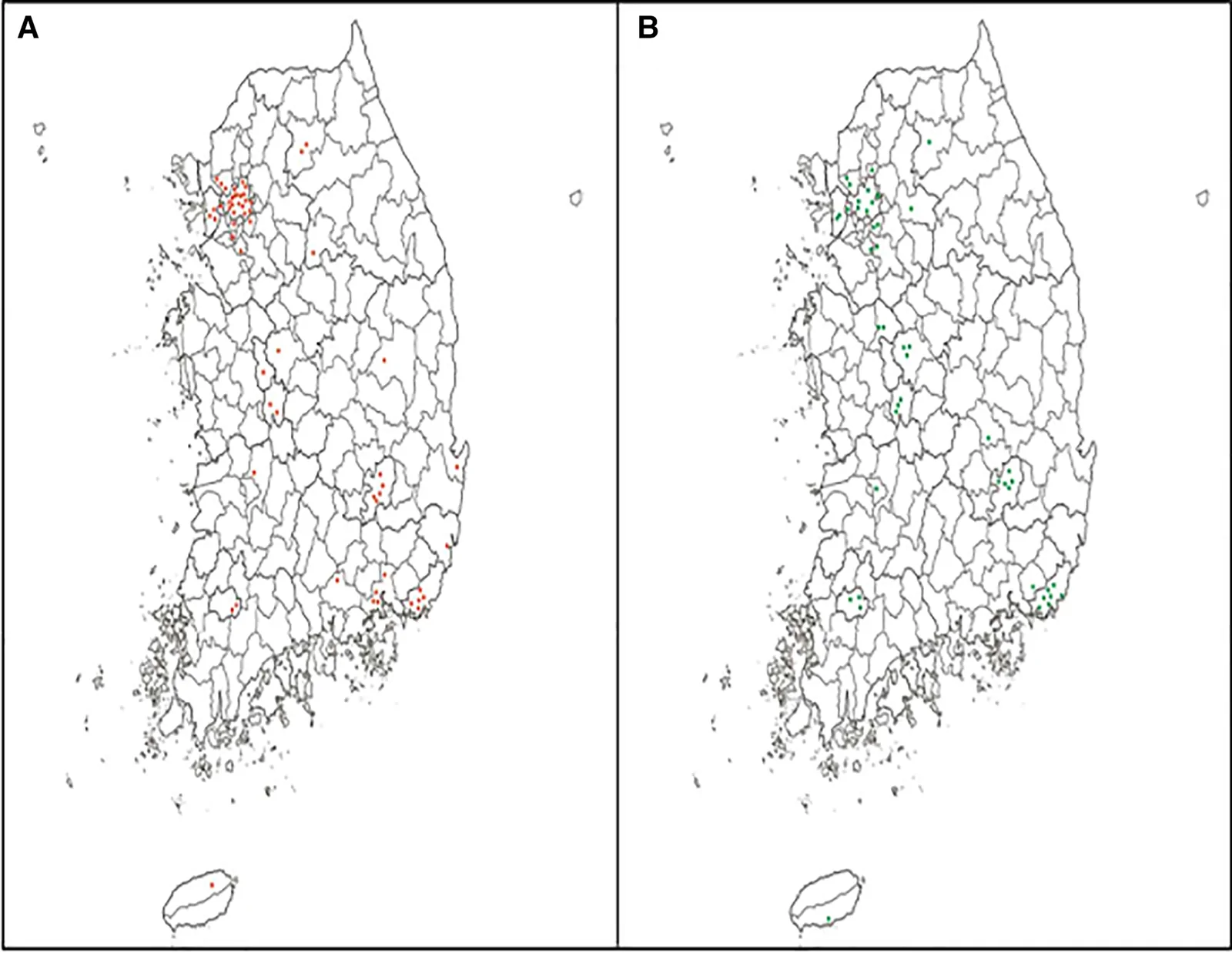
Geographical distribution of specialized stroke and rehabilitation medical infrastructure in South Korea. (A) Distribution of stroke treatment rooms. (B) Distribution of convalescent rehabilitation medical institutions.

**Figure 3.**
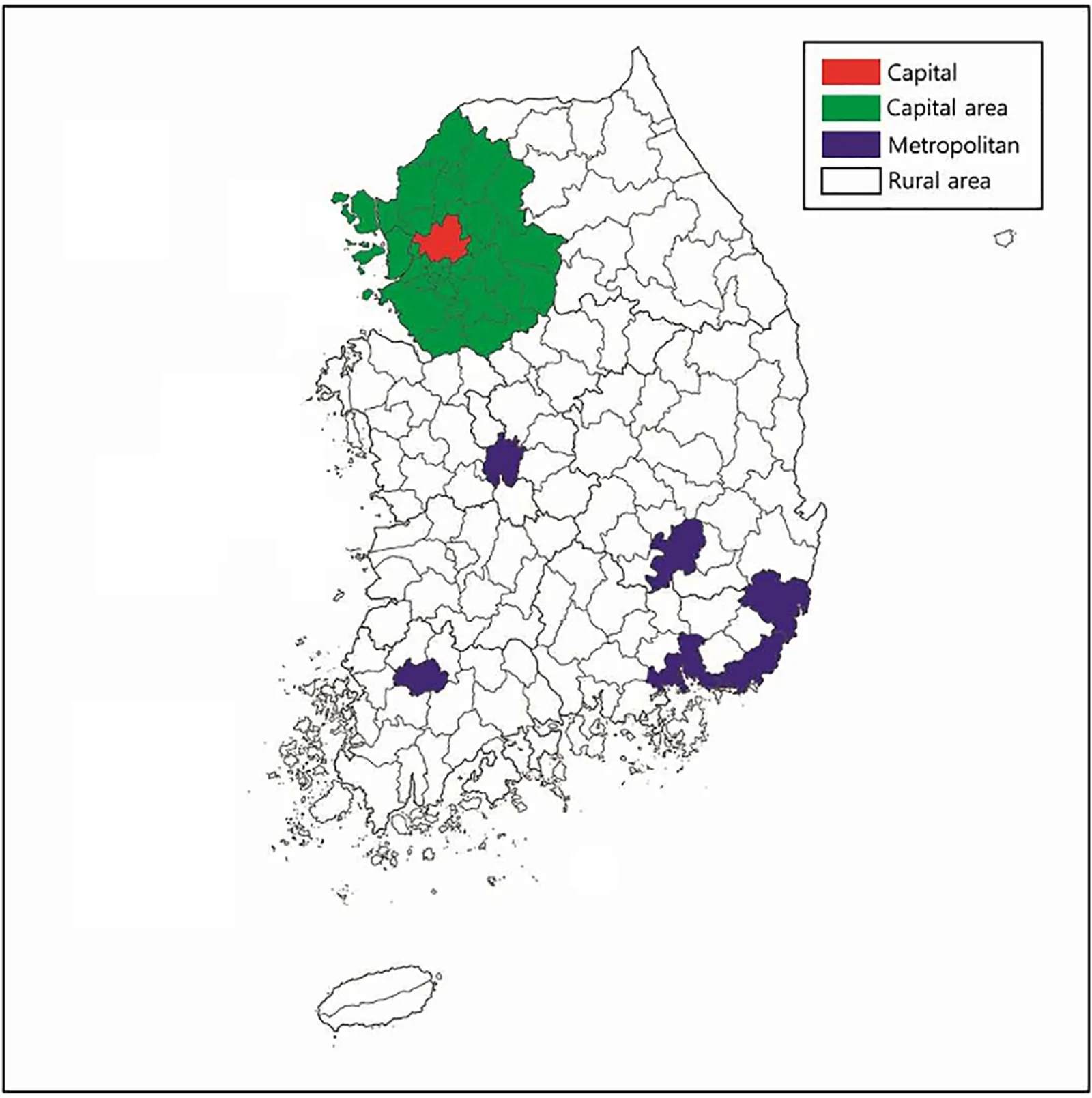
Categorization of study regions into 4 geographic categories based on administrative divisions, population size, and medical infrastructure density: Capital (Seoul), Capital area (Incheon and Gyeonggi-do), Metropolitan (major cities outside the capital area with a population ≥ 1 million), and Rural area (all other regions).

### 2.4. Statistical analysis

Statistical analyses were performed in several stages. We first compared the baseline demographic, socio-economic, and clinical characteristics of patients according to mortality status using the Chi-square test. To investigate the disparity in healthcare utilization, a logistic regression model was employed to analyze the association between the region of residence and the utilization of PT and OT services among stroke patients, calculating ORs and 95% confidence interval (CIs). Furthermore, to distinguish between patients requiring intensive rehabilitation and those with different clinical severity levels, we analyzed the study population by dividing them into inpatient and outpatient groups. Survival outcomes based on these classifications were compared using Kaplan–Meier survival curves. Finally, a Cox proportional hazards model was used to estimate hazard ratios (HRs) and 95% CIs to identify factors affecting long-term survival; specifically, given that accessibility outcomes were comparable across major urban centers, the primary Cox model focused on contrasting the Capital and rural areas to evaluate the clinical impact of extreme geographic disparities without diluting outcome signals. In the multivariate models, results were adjusted for sex, age, income level, Charlson Comorbidity Index (CCI) scores, social security type, and disability level. All statistical significance was set at *P* < .05. Analyses were conducted using SAS software, version 9.4 (SAS Institute).

## 3. Results

### 3.1. Sociodemographic characteristics and mortality

Table 1 summarizes the sociodemographic characteristics of the 16,804 patients diagnosed with stroke (males, 8627 [51.3%]; females, 8177 [48.7%]). The mortality rate demonstrated a marked age-dependent increase, rising from 7.3% in patients under 40 years to 70.8% in those over 70 years. Furthermore, socioeconomic disparities were evident; the lowest income quartile and Medical Aid recipients exhibited significantly higher mortality rates compared to their respective counterparts (all *P <* .001). Similarly, the presence of a disability and higher CCI scores were positively correlated with increased mortality (*P <* .001).

**Table 1:** Demographic and sociological characteristics of the participants.

	Study population		Survivors		Non-survivors		*P*-value
	n	%	n	%	n	%	
Total	16,804	100	8784	52.3	8020	47.7	
Sex							.0433
Male	8627	51.3	4575	53.0	4052	47.0	
Female	8177	48.7	4209	51.5	3968	48.5	
Age (yrs)							<.001
<40	537	3.2	498	92.7	39	7.3	
40–49	1394	8.3	1212	86.9	182	13.1	
50–59	2927	17.4	2351	80.3	576	19.7	
60–69	4032	24.0	2408	59.7	1624	40.3	
≥70	7914	47.1	2315	29.3	5599	70.8	
Income level							<.001
1 (lowest quartile)	4278	25.5	1907	44.6	2371	55.4	
2	3327	19.8	1972	59.7	1355	40.7	
3	4520	26.9	2531	56.0	1989	44.0	
4	4679	27.8	2374	50.7	2305	49.7	
Social security type							<.001
Workplace subscribers	9266	55.1	4945	53.8	4321	46.6	
Local subscribers	5761	34.3	3248	56.4	5761	43.6	
Medical aids	1777	10.6	591	33.3	1186	66.7	
Disability status							<.001
Normal	14,152	84.2	7784	54.8	6404	45.3	
Mild	732	4.4	224	30.6	508	69.4	
Severe	1920	11.4	812	42.3	1108	57.7	
CCI score							<.001
0	10,055	59.8	5692	56.6	4363	43.4	
1	2281	27.3	2281	49.8	2300	50.2	
≥2	811	12.9	811	37.4	1357	62.6	
Type of region							<.001
Capital	2680	16.0	1567	58.5	1113	41.5	
Capital area	3875	23.0	2090	53.9	1785	46.1	
Metropolitan	3513	20.9	1882	53.6	1631	46.4	
Rural area	6736	40.1	3245	48.2	3491	51.8	

CCI = Charlson Comorbidity Index.

### 3.2. Regional disparities in rehabilitation accessibility

In the multivariate logistic regression analysis (Table 2), rural area remained an independent predictor of limited access to rehabilitation services. Specifically, rural area was associated with a lower likelihood of being prescribed PT (ORs = 0.889; 95% CIs, 0.809–0.978; *P* = .029). This disparity was even more pronounced for OT, where rural patients had a significantly lower probability of prescription compared to their urban counterparts (ORs = 0.802; 95% CIs, 0.726–0.886; *P* < .001). Furthermore, the odds of receiving combined rehabilitation (both PT and OT) were significantly reduced for patients living in rural area (ORs = 0.884; 95% CIs, 0.805–0.971; *P* = .015).

**Table 2:** Regional disparities in rehabilitation accessibility: Odds ratios (ORs) and 95% confidence intervals (CIs) from multiple logistic regression analysis.

	PT	OT	PT and OT
	ORs (95% CIs)	ORs (95% CIs)	ORs (95% CIs)
Capital	1 (Ref.)	1 (Ref.)	1 (Ref.)
Capital area	0.965 (0.871–1.07)	0.962 (0.864–1.071)	0.962 (0.869–1.066)
Metropolitan	1.010 (0.909–1.122)	1.033 (0.926–1.153)	1.019 (0.918–1.131)
Rural area	0.889 (0.809–0.978)*	0.802 (0.726–0.886)*	0.884 (0.805–0.971)*

Results were obtained by adjusting for sex, age, income level, Charlson Comorbidity Index, social security type, and disability level.

OT = occupational therapy, PT = physical therapy, Ref. = reference.

* *P* < .05.

In contrast, patients residing in the Capital area and Metropolitan regions showed no statistically significant differences in rehabilitation accessibility compared to those in the Capital across all prescription modalities, including single PT, single OT, and combined rehabilitation (all *P* > .05; e.g., for combined PT and OT, Capital area: ORs = 0.962, 95% CIs, 0.869–1.066, *P* = .45; Metropolitan: ORs = 1.019, 95% CIs, 0.918–1.131, *P* = .72).

### 3.3. Impact of regional disparities on long-term mortality

To evaluate the long-term consequences of regional resource imbalances without diluting the primary outcome signal, the multivariable Cox regression analysis specifically examined the risk of 10-year mortality by comparing Rural area with the Capital (Table 3). Kaplan–Meier survival curves demonstrated a significant difference in long-term cumulative survival between regions among patients receiving inpatient rehabilitation, with the survival gap progressively widening over the 10-year follow-up period (*P* = .024; Fig. 4A). Specifically, rural area was independently associated with a significantly higher risk of 10-year mortality compared to the Capital in the inpatient rehabilitation group (HRs = 1.288; 95% CIs, 1.015–1.635; *P* = .037).

**Table 3:** Cox proportional hazards regression analysis for 10-year mortality according to rehabilitation setting and region.

	HRs (95% CIs)	*P*-value
Inpatient		
Capital	1 (Ref.)	
Rural area	1.288 (1.015–1.635)	.037
Outpatient		
Capital	1 (Ref.)	
Rural area	1.011 (0.917–1.115)	.820

Results were obtained by adjusting for sex, age, income level, Charlson Comorbidity Index, social security type, and disability level.

CIs = confidence interval, HRs = hazard ratio, Ref. = reference.

**Figure 4.**
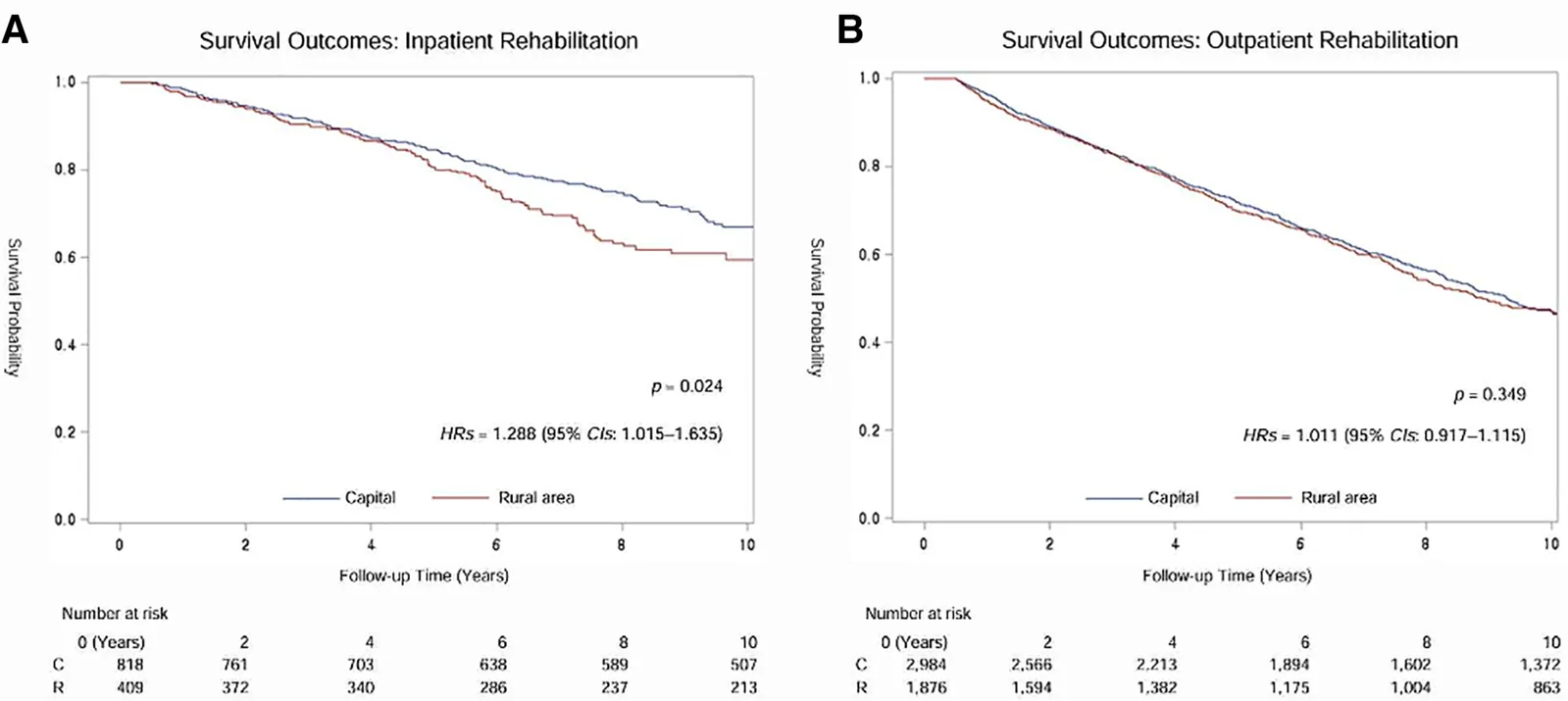
Kaplan–Meier survival curves for 10-year cumulative survival rate according to rehabilitation setting across geographic regions. (A) Inpatient rehabilitation group. (B) Outpatient rehabilitation group. CIs = confidence intervals, HRs = hazard ratios.

In contrast, no statistically significant regional differences were observed in cumulative survival (*P* = .349; Fig. 4B) or overall 10-year mortality within the outpatient rehabilitation group (HRs = 1.011; 95% CIs, 0.917–1.115; *P* = .820).

## 4. Discussion

This study utilized the NHIS-NSC database to analyze the impact of rehabilitation services on stroke survival, while controlling for various demographic and clinical factors. The most critical finding of this study is that even after strictly adjusting for individual variables, regional disparity remained a potent and independent predictor of mortality. Our results reveal a critical dual-barrier for stroke survivors in rural area: significantly limited access to rehabilitation services and poorer survival outcomes following inpatient treatment.

Stroke leads to various functional impairments, including physical disability, cognitive deficits, and dysphagia.^[6]^ If patients do not receive intensive intervention during the early stage, it can cause irreversible functional decline; however, timely rehabilitation helps prevent complications and promotes recovery.^[23,24]^ According to the study by Yoo et al,^[5]^ intensive rehabilitation is a crucial determinant of long-term survival in stroke patients. The present study expands upon those findings by identifying that the “survival benefit” of such intensive treatment is heavily compromised by geographic location. Our multiple logistic regression analysis (Table 2) reveals a fundamental barrier: patients in rural areas were significantly less likely to receive PT (ORs = 0.889), OT (ORs = 0.802), or both (PT and OT; ORs = 0.884) compared to those in the capital. This 11 to 20% reduction in therapy access represents a critical population-level disparity, as timely physical and occupational rehabilitation are key determinants of poststroke functional recovery. These findings align closely with previous national and international studies reporting a 10 to 25% lower likelihood of rural stroke survivors accessing specialized rehabilitation services.^[25]^ While accessibility in Metropolitan remained comparable to Capital due to regional tertiary centers, rural area faced a systemic bottleneck in early rehabilitation delivery.

The consequences are even more evident in the long-term prognosis. For patients receiving Inpatient Rehabilitation, residing in a rural area remained a potent independent risk factor for mortality, with an adjusted HRs of 1.288 (95% CIs, 1.015–1.635, *P* = .037; Table 3). The Kaplan–Meier curves further illustrated this widening survival gap over the 10-year follow-up period (*P* = .024; Fig. 4A).

This progressive divergence suggests that the lack of timely, intensive inpatient rehabilitation in rural area leads to unrecovered functional deficits and secondary medical complications, which continuously impair long-term survival. This finding aligns with prior studies conducted in other countries, such as the United States and Taiwan,^[12,26]^ which similarly reported that regional disparities in inpatient rehabilitation accessibility significantly contribute to long-term poststroke mortality.

In contrast, no such disparity was found in the outpatient setting (HRs = 1.011; *P* = .820; Fig. 4B). This distinction emphasizes that once patients stabilize and transition to outpatient care, survival outcomes are relatively homogenized across regions. Therefore, the long-term mortality gap is predominantly driven by structural deficits during the acute and subacute inpatient phases, where immediate multidisciplinary care is most critical. Furthermore, the lack of significant mortality divergence across major metropolitan areas aligns with their comparable rehabilitation accessibility, confirming that long-term survival disparities are selectively concentrated in resource-scarce rural regions.

These disparities are fundamentally rooted in the geographical maldistribution of healthcare resources. This structural inequality is starkly illustrated by the high concentration of both stroke care units (Fig. 2A) and convalescent rehabilitation hospitals (Fig. 2B) within Seoul. This centralization of infrastructure naturally draws the healthcare workforce, as evidenced by the fact that over 62% of physical therapists and 66% of occupational therapists in South Korea are concentrated in metropolitan areas.^[27]^ Consequently, structural disparities in working environments and professional opportunities drive a persistent concentration of therapists in urban centers, creating a chronic labor supply bottleneck that further exacerbates the service gap in the rural area.^[28]^

Ultimately, fostering healthcare equity requires more than just resource distribution; it demands a fundamental shift toward ensuring that the survival benefit of intensive inpatient rehabilitations is accessible to all, regardless of their residence. To achieve this, a dual strategy backed by dedicated financial and economic mechanisms is essential: allocating targeted budgetary support to expand and enhance the clinical capacities of regional medical centers for high-quality early-stage care, and implementing robust financial incentives – such as regional healthcare subsidies and loan forgiveness programs – to ensure skilled therapists remain rooted within the rural area. Only through such economically supported, integrated efforts can we bridge the geographical gap in stroke survival and guarantee a fair chance at recovery for every patient.

### 4.1. Study limitations

This study has several limitations. First, while we redefined the regions into 4 categories to reflect the strategic distribution of stroke units and convalescent rehabilitation hospitals, this classification might not perfectly align with the administrative districts defined by the Korean Local Autonomy Act. Second, the NHIS-NSC database lacks specific clinical stroke severity scores (e.g., NIHSS) and functional status (e.g., mRS). To mitigate this, we followed the methodology of previous research by including disability levels and the CCI as proxy measures for severity, yet some residual confounding may persist. Third, as this study was conducted within the South Korean healthcare context, the findings may have limited generalizability to other countries with different medical systems.

Despite these limitations, this study has distinct strengths: it utilizes objective, nationwide data on both PT and OT participation to comprehensively evaluate regional disparities across Capital, Capital area, Metropolitan, and Rural area classifications.

## 5. Conclusion

In conclusion, this nationwide cohort study demonstrates a critical regional disparity in poststroke rehabilitation: while outcomes in Capital area and Metropolitan remain comparable to Capital, stroke survivors in Rural area face significant barriers in accessing early rehabilitation services and experience poorer long-term survival following inpatient care. Crucially, once patients stabilize and transition to outpatient settings, regional survival gaps disappear, proving that mortality disparities are primarily driven by structural bottlenecks during acute and subacute inpatient phases. Policy interventions must focus on bridging these early inpatient resource gaps and retaining skilled rehabilitation workforce in Rural area to ensure healthcare equity. Future studies should evaluate the long-term functional and economic impacts of these policy interventions to guarantee equitable poststroke recovery nationwide.

## Acknowledgments

This study use NHIS-NSC data (NHIS-2022-2-116). The authors would like to thank the National Health Insurance Service for providing the data.

## Author contributions

**Data curation:** Jung-Kyu Choi.

**Funding acquisition:** Dong-Yup Yoo.

**Investigation:** Dong-Yup Yoo.

**Methodology:** Dong-Yup Yoo.

**Software:** Chang-Yoon Baek.

**Supervision:** Jung-Kyu Choi, Chang-Yoon Baek.

**Writing – original draft:** Dong-Yup Yoo.

**Writing – review & editing:** Dong-Yup Yoo.
